# 1316. Clinical Utility of Pharmacogenomic Testing in Refractory Coccidioidal Meningitis

**DOI:** 10.1093/ofid/ofad500.1155

**Published:** 2023-11-27

**Authors:** Janis E Blair, Adrijana Kekic, Marie Grill

**Affiliations:** Mayo Clinic Hospital, Phoenix, Arizona; Mayo Clinic, Phoenix, Arizona; Mayo Clinic AZ, Scottsdale, Arizona

## Abstract

**Background:**

Coccidioidomycosis is an endemic fungal infection in the desert southwestern USA. Less than 1% of infections are complicated by coccidioidal meningitis (CM), which requires lifetime treatment, and has high morbidity. IDSA guidelines recommend high dose fluconazole (flu) as first line treatment, but up to 31% of CM patients (pts) fail flu. Guidelines have not yet articulated best subsequent therapy, but options include itraconazole (itra), voriconazole (vori), posaconazole and isavuconazole (isa). Excellent adherence to medications is imperative, and therapeutic fluconazole (flu) serum levels correlate with successful CM outcome. Many azoles are substrates of P450/CYP enzymes, and genetic alterations may influence medication levels. We have observed many CM pts who swear to adherence yet have low therapeutic azole levels. Our aim was to summarize our experience with pharmacogenomic (PGx) testing to guide treatment of CM.

**Methods:**

We conducted a retrospective review of CM pts in our Coccidioidomycosis Clinic who underwent 1 of 2 targeted gene queries, either an internal 9-gene query or an external vendor 27 gene test. A PGx pharmacist interpreted results.

**Results:**

From 1/1/2021 – 4/30/2023, we identified 6 pts with poorly controlled CM as manifested by repeated hospitalizations, progressively abnormal findings on lumbar puncture and/or magnetic resonance imaging of the central nervous system. Among the 6, 3 (50%) were male, 5 (83%) white, mean age 45 (28-71) years, with 34-month mean duration of illness. None were immunosuppressed. CM was complicated by strokes in 3, and hydrocephalus requiring ventriculoperitoneal shunt in 2. Five failed initial high dose flu and most had some low therapeutic levels without other medications acting as CYP inducers/inhibitors. 3 pts had the 9 gene test and 3 had the 27 gene test. Clinically relevant PGx abnormalities were identified in 5, including variants of CYP 2C19, 2C9, 3A4 and 3A5, which confer altered metabolism of vori, itra and isa, respectively. Following PGx, treatment was adjusted in 4 pts.

**Conclusion:**

PGx testing is a potential adjunctive tool to avoid ineffective therapies or medication toxicity in an infection whose treatment success strongly depends on consistent therapeutic medication levels over a lifetime.

**Disclosures:**

**All Authors**: No reported disclosures

